# Gymnotic Delivery of LNA Mixmers Targeting Viral SREs Induces HIV-1 mRNA Degradation

**DOI:** 10.3390/ijms20051088

**Published:** 2019-03-03

**Authors:** Frank Hillebrand, Philipp Niklas Ostermann, Lisa Müller, Daniel Degrandi, Steffen Erkelenz, Marek Widera, Klaus Pfeffer, Heiner Schaal

**Affiliations:** 1 Institute of Virology, Medical Faculty, Heinrich-Heine-University Düsseldorf, 40225 Düsseldorf, Germany; Frank.Hillebrand@med.uni-duesseldorf.de (F.H.); Philipp.Ostermann@uni-duesseldorf.de (P.N.O.); Lisa_Muller@dfci.harvard.edu (L.M.); Steffen.Erkelenz@gmx.de (S.E.); Marek.Widera@uni-due.de (M.W.); 2 Institute of Medical Microbiology and Hospital Hygiene, Medical Faculty, Heinrich-Heine-University Düsseldorf, 40225 Düsseldorf, Germany; Daniel.Degrandi@uni-duesseldorf.de (D.D.); Klaus.Pfeffer@med.uni-duesseldorf.de (K.P.)

**Keywords:** antisense oligonucleotides, locked nucleic acids, splicing regulatory elements, mRNA degradation, human immunodeficiency virus type 1 (HIV-1)

## Abstract

Transcription of the HIV-1 provirus generates a viral pre-mRNA, which is alternatively spliced into more than 50 HIV-1 mRNAs encoding all viral proteins. Regulation of viral alternative splice site usage includes the presence of splicing regulatory elements (SREs) which can dramatically impact RNA expression and HIV-1 replication when mutated. Recently, we were able to show that two viral SREs, G_I3_-2 and ESE_tat_, are important players in the generation of viral *vif*, *vpr* and *tat* mRNAs. Furthermore, we demonstrated that masking these SREs by transfected locked nucleic acid (LNA) mixmers affect the viral splicing pattern and viral particle production. With regard to the development of future therapeutic LNA mixmer-based antiretroviral approaches, we delivered the G_I3_-2 and the ESE_tat_ LNA mixmers “nakedly”, without the use of transfection reagents (gymnosis) into HIV-1 infected cells. Surprisingly, we observed that gymnotically-delivered LNA mixmers accumulated in the cytoplasm, and seemed to co-localize with GW bodies and induced degradation of mRNAs containing their LNA target sequence. The G_I3_-2 and the ESE_tat_ LNA-mediated RNA degradation resulted in abrogation of viral replication in HIV-1 infected Jurkat and PM1 cells as well as in PBMCs.

## 1. Introduction

According to the World Health Organization (WHO) in 2017, 36.9 million people were living with the human immunodeficiency virus type 1 (HIV-1) and globally only 21.7 million people received antiretroviral therapy (ART) which combines drugs targeting crucial steps of the HIV-1 replication cycle. However, although ART successfully reduces viral replication to a level below the detection limit, and thus saves the lives of many HIV-infected individuals, it is not curative, and non-adherent patients are especially at risk of developing multidrug-resistant viruses.

Furthermore, life-long ART is often accompanied by multiple adverse side-effects (e.g., lipodystrophy, insulin resistance, dyslipidemia, chronic inflammation) [1,2]. Thus, the identification of additional and alternative targets within the viral life cycle for antiviral drug development is desirable.

As a member of the family of retroviruses (retroviridae) the (+) RNA genome of HIV-1 is reverse transcribed, imported into the nucleus and integrated into the host cellular genome. After transcription the viral pre-mRNA undergoes extensive alternative splicing leading to viral mRNA transcripts essential for all viral proteins. The viral mRNAs are grouped into three viral RNA classes according to their size: The spliced and intron-less 2 kb class (including *tat*, *rev* and *nef* mRNAs), the spliced but intron-containing 4 kb class (including *vif, vpr, vpu* and *env* mRNAs), and the unspliced 9 kb class which serves as mRNA for Gag/Pol and as the viral genome [3]. Generation of more than 50 alternatively spliced viral mRNAs is regulated by differential usage of at least five viral splice donor sites (5′-splice sites) and eight viral splice acceptor sites (3′-splice sites) as well as the presence of several viral exonic and intronic splicing regulatory elements (SREs) [4,5,6,7,8]. Here, the SREs are bound by family members of the serine- and arginine-rich phosphoproteins (SR proteins) or heterogeneous nuclear ribonucleoproteins (hnRNPs), which positively or negatively influence viral splice site selection depending on their position relative to them [5,8,9]. For efficient replication a balanced generation of all viral mRNAs is crucial. Therefore, disruption of the viral splicing process, e.g., by preventing binding of splicing regulatory proteins to their RNA target seems to be a promising approach to impair viral replication. Indeed, as shown in several mutational analyses of viral SREs, interference with the SREs’ function not only dramatically impacts viral splicing or RNA expression, but also influences HIV-1 particle production [4,10,11,12,13,14,15,16].

Masking viral sequences, e.g., to prevent protein binding, however, has been tried already in the late 1980s when two groups provided evidence that HIV-1 replication can be reduced by adding DNA-antisense oligonucleotides (ASOs), complementary to HIV-1 RNA sequences, to cell culture medium. At that time, unfortunately, very high ASO concentrations were necessary in order to impair HIV-1 RNA expression [17,18], hampering the dissemination of this approach. Later on, mostly by transfection or cell-free in vitro experiments, it was demonstrated that masking various HIV-1 sequences such as the HIV-1 dimerization initiation site, the trans-activation response element (TAR) element, the major splice donor 1 or the viral guanine-adenine-rich (GAR) SRE by ASOs or modified U7 snRNAs, also interfered with viral RNA expression [19,20,21,22]. The applied ASOs, however, have to exhibit obligatory characteristics: (i) Efficient masking and specific binding of the target sequence, (ii) low toxicity range, and (iii) an increased stability against endo- and exonucleases.

In the past, several ASOs exhibiting these characteristics have been generated. These include, e.g., 2′-*O*-methyl (2′-OMe) and 2′-*O*-methoxyethyl (2′-MOE) ASOs, as well as phosphorodiamidate morpholinos (PMOs). All these ASOs exhibit either a modified phosphate-backbone (e.g., phosphorothioate (PS) backbone), and modifications at the 2′ sugar position within their ribose moiety or a morpholine ring instead of the furanose ring (PMOs). Furthermore, because these mentioned ASOs can influence alternative splice site recognition when targeted against SREs or splice sites, they are also applied as so-called splice-switching oligonucleotides (SSOs) [23,24]. Indeed, already two FDA-approved antisense oligonucleotides were developed targeting either the exon 51 of the dystrophin pre-mRNA for exon skipping (a PMO ASO, Eteplirsen/Exondys 51), or an intronic SRE inducing exon 7 inclusion in the survival motor neuron protein 1 and 2 mRNAs (a 2′-MOE ASO, Nusinersen/Spinraza). Both ASO strategies are promising therapeutics for patients suffering from Duchenne muscular dystrophy (DMD) or spinal muscular atrophy (SMA) [24,25,26,27].

In addition to the aforementioned ASOs, locked nucleic acids (LNAs) are also promising and commonly used ASOs, which can either be used as SSOs to induce steric blocks (LNA mixmers, a combination of LNA and DNA residues), or to induce degradation of target mRNAs (LNA gapmers, with LNAs at the 5′- and 3′-end, and a DNA strand in the center of the ASO). Due to an extra methylene bridge between the 2′-O- and the 4′-C-atoms of the ribose moiety, LNAs are locked in the ideal conformation for Watson–Crick binding, resulting in an increased melting temperature and an extreme antisense-target-duplex stability [28,29].

Furthermore, it was shown that LNAs can be administered without the use of transfection reagents in a process termed “gymnosis” and that human primary T-cells are susceptible to gymnotic LNA delivery [30,31,32]. For gymnotic cellular uptake a phosphorothioate- (PS) backbone is essential and therefore, the PS modification must be present in at least 75% of all phosphate linkage in ASOs like 2′-OMe, 2′-MOE or LNAs [33]. ASOs containing a PS-backbone enter the cells via a combination of adsorptive and fluid-phase endocytosis and later accumulate in multivesicular bodies, late endosomes and lysosomes. However, how LNAs escape these membrane vehicles to fulfil their purpose is poorly understood, and is still under investigation [34,35,36,37]. LNAs also have an increased stability against endo- and exonucleases, and are in a low toxicity range, hence displaying all characteristics for *in vivo* usage. Moreover, LNA compounds displaying good pharmacokinetics and –dynamics have been used as “naked” phosphorothioate-modified oligonucleotides, and have been successfully tested in many animal model systems (e.g., mice, rats, monkeys, chimpanzees) and humans [28,29]. Miravirsen for example, an LNA compound developed by the pharmaceutical company Santaris Pharma A/S, is currently in phase II of clinical trials, and masks the liver-specific micro-RNA miR-122. This LNA-based drug is a therapeutic agent for hepatitis C virus (HCV) infection because miR-122 serves as a crucial host factor for HCV [24,38,39].

Recently, we have shown that both splicing regulatory elements G_I3_-2 and ESE_tat_ play a major role in the generation of viral mRNA species such as *vif*, *vpr* and *tat* mRNAs. Furthermore, we demonstrated that mutating these elements by site-directed mutagenesis or masking these elements by co-transfecting host cells with the proviral DNA and the respective LNA mixmer successfully interfered with viral pre-mRNA splicing and viral replication [13,15]. With regard to the development of an alternative antiretroviral therapy we gymnotically-delivered the G_I3_-2 and ESE_tat_ LNA mixmers into HIV-1 infected cells and observed that “nakedly” delivered LNA mixmers localize within the cytoplasm, and induce degradation of viral mRNA species containing their target sequence rather than influencing recognition of adjacent splice sites. Consequently, gymnotically-delivered LNA mixmers efficiently block viral replication in HIV 1 infected Jurkat and PM1 cells as well as in PBMCs demonstrating their antiretroviral potential.

## 2. Results

### 2.1. Gymnotically-Delivered LNA Mixmers Binding the SREs G_I3_-2 and ESE_tat_ Specifically Induce Degradation of Their Target mRNAs

**Figure 1:**
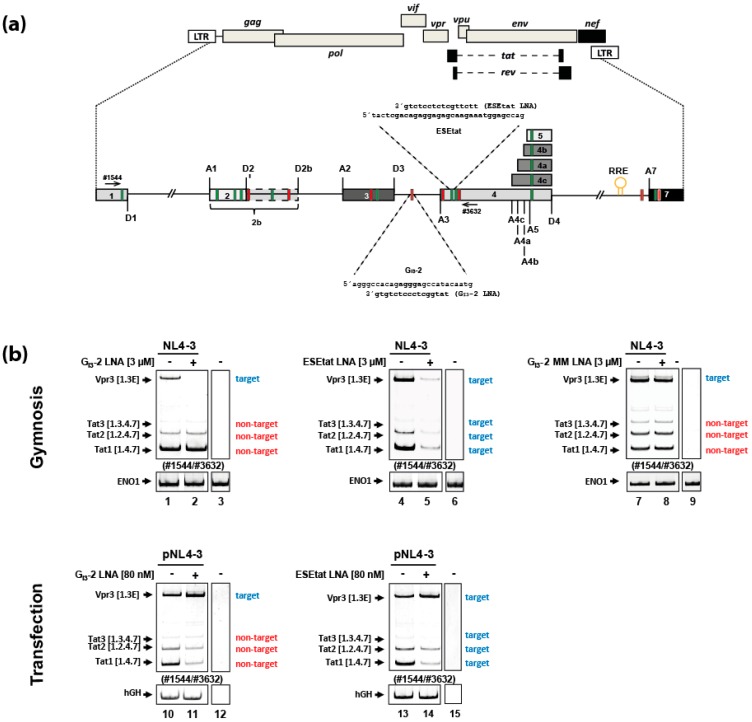
Gymnotically-delivered LNA mixmers targeting viral splicing regulatory elements (SREs) induce degradation of viral mRNAs containing their target sequence. (**a**) The diagram illustrates the HIV-1 proviral genome including open reading frames (ORFs) of viral proteins, long terminal repeats (LTRs), viral splice donor (D) and acceptor (A) sites as well as the viral splicing regulatory elements (enhancing SREs: Green, silencing SREs: Red). The Rev responsive element (RRE) for the Rev-dependent export of the intron-containing viral mRNA species is indicated by an open circle within the last HIV-1 intron. The primer binding sites (#1544, #3632; see Figure 1a) are also shown. The sequences as well as their target sequences of the G_I3_-2 and ESE_tat_ LNAs are indicated. (**b**) Upper panel: Jurkat cells were infected with NL4-3 (MOI: 0.005) and the LNA mixmers were gymnotically-delivered by adding them at a concentration of 3 µM to the cell culture medium 6 h post infection. Cellular ENO1 was detected to monitor RT-PCR efficiency. Lower panel: HeLa cells were co-transfected with pNL4-3 and either the G_I3_-2 or ESE_tat_ LNA mixmer. To monitor transfection and RT-PCR efficiencies, a plasmid expressing human growth hormone 1 (hGH) was co-transfected and hGH mRNA was detected using specific primers. Blue: Viral LNA target mRNAs; red: Viral LNA non-target mRNAs. HIV-1 mRNA species are indicated to the left of the gel images according to the nomenclature published previously [3]. Lanes 3, 6, 9, 12 and 15 represent mock samples running on the same gel. MM: Mismatch.

Both viral splicing regulatory elements (SREs), G_I3_-2 and ESE_tat_ (Figure 1a), localized within HIV-1 intron 3 and downstream of the viral SA3 respectively, are involved in regulating HIV-1 splice site usage which is essential for the generation of *tat* as well as *vpr* and *vif* mRNA species. In previous studies we were able to show that individual delivery of either locked nucleic acid (LNA) mixmer, masking the G_I3_-2 or the ESE_tat_ element (Figure 1a), by transfection induced changes within the viral splicing pattern comparable to their mutational inactivation. Likewise, they also efficiently interfered with HIV-1 replication and RNA expression [13,15].

To test if the G_I3_-2 and ESE_tat_ LNAs (Figure 1a) also affect the splicing pattern when delivered “nakedly” (via gymnosis) we added the LNAs (3 µM) to the culture medium of HIV-1 infected (laboratory strain NL4-3, MOI: 0.005) Jurkat cells without the use of any transfection reagent. To be able to compare the effect of the gymnotically-delivered LNA mixmers on the viral mRNA splicing pattern, we also transfected HeLa cells with both LNA mixmers (80 nM) as described in [13,15]. Twenty-four hours after LNA addition we isolated total RNA and performed RT-PCR analysis and investigated the viral splicing pattern.

After gymnotic delivery of the G_I3_-2 or the ESE_tat_ LNA mixmers to HIV-1 infected Jurkat cells, both LNAs specifically induced a decrease only in their targeted transcript isoforms: for the G_I3_-2 LNA mixmer, the *vpr*3 mRNA (Figure 1b, cf. lanes 1 and 2 [upper panels]) and for the ESE_tat_ LNA mixmer the *vpr3*, *tat1*, *tat2* and *tat3* mRNAs (Figure 1b cf. lanes 4 and 5 [upper panels]). This result was in contrast to the previously described effect [13,15] of both delivered LNAs by transfection, which induced, comparable to the corresponding mutational inactivation of the G_I3_-2 or ESE_tat_ SREs, an increase of *vpr* mRNA and reduced *tat* mRNA level (Figure 1b, cf. lanes 10 and 11 and lanes 13 and 14 [lower panels]; [13,15]). Because the *vpr3* mRNA contains the LNA target sequence for both LNA mixmers whereas the *tat* mRNAs only contain the LNA target sequence for the ESE_tat_ LNA (Figure 1b, cf. target vs. non-target) the impact of both gymnotically-delivered LNA mixmers on the viral mRNA expression indicated an LNA mixmer-induced degradation of their target mRNAs rather than affecting pre-mRNA splicing (Figure 1b, cf. target vs. non-target). A mismatch (MM) G_I3_-2 LNA, which neither affected the expression of the *vpr3* mRNA nor the *tat* mRNAs (Figure 1b. cf. lanes 1 and 2 with lanes 7 and 8 [upper panels]) demonstrated its sequence-specificity.

### 2.2. A Gymnotically-Delivered SRSF6 Exon/Junction LNA Mixmer Induces Degradation of the SRSF6 mRNA

**Figure 2:**
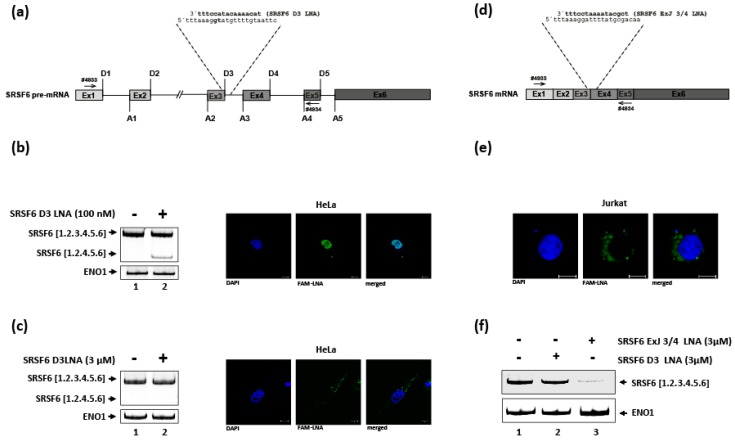
Influence of 6-FAM™-SRSF6 LNA mixmers on SRSF6 expression and their cellular localization. (**a**) Schematic drawing of the SRSF6 pre-mRNA with the indicated 6-FAM™-SRSF6 D3 LNA sequence as well as its target sequence and primer binding sites. (**b**) HeLa cells were transfected with the 6-FAM™-SRSF6 D3 LNA and after 24 h either total RNA was isolated to perform a RT-PCR (left panel), or the cells were fixed and the LNAs cellular localization was analyzed using confocal laser scanning microscopy (right panel; white bar: 10 µm). (**c**) The 6-FAM™-SRSF6 D3 LNA (3 µM) was gymnotically-delivered into HeLa cells, and after 48 h incubation, either total RNA was isolated (RT-PCR, left panel), or the cells were analyzed via confocal laser scanning microscopy (right panel; white bar: 10 µm). (**d**) Schematic drawing of the SRSF6 mRNA with the indicated 6-FAM™-SRSF6 ExJ 3/4 LNA sequence as well as its target sequence and primer binding sites. (**e**) Cellular localization of the 6-FAM™-SRSF6 ExJ 3/4 LNA in Jurkat cells after gymnotic delivery (white bar: 5 µm). (**f**) RT-PCR analysis after gymnotic delivery of the 6-FAM™-SRSF6 D3 and 6-FAM™-SRSF6 ExJ 3/4 LNA into Jurkat cells. Total RNA was isolated 48 h after LNA delivery.

To rule out that the LNA-mediated mode of action on RNA expression was only specific for LNAs directed against HIV-1 RNA, we tested an additional LNA mixmer targeting the cellular serine/arginine-rich splicing factor 6 (SRSF6) mRNA. Here, to analyze the impact of the LNA mixmer (SRSF6 D3 LNA) on SRSF6 splice donor usage, the LNA was complementary designed to mask the splice donor 3 (D3) sequence of the SRSF6 pre-mRNA (Figure 2a). Furthermore, the LNA mixmer was 5′-labeled with a 6-Carboxyfluorescein (6-FAM™) modification to allow analysis of its cellular localization after delivery by using confocal laser scanning microscopy.

First, as reference, we delivered the SRSF6 D3 LNA mixmer by transfecting HeLa cells followed by RT-PCR analysis. As expected, masking splice donor 3 competed with U1 snRNA binding, and thus led to SRSF6 exon 3 skipping (Figure 2b, cf. lanes 1 and 2). Moreover, a predominantly nuclear localization of the SRSF6 D3 LNA mixmer could be observed (Figure 2b, right panel).

In contrast, when the SRSF6 D3 LNA was delivered gymnotically, skipping of exon 3 could not be detected (Figure 2c, cf. lanes 1 and 2). Furthermore, when analyzing the intracellular localization of the “nakedly” delivered SRSF6 D3 LNA mixmer, we detected fluorescence signals exclusively in the cytoplasm within a speckled distribution (Figure 2c, right panel). Because splicing takes place in the nucleus, this result plausibly explains why we could not detect any effects on SRSF6 exon 3 inclusion after gymnotic LNA delivery. Furthermore, LNA-mediated RNA degradation of the SRSF6 mRNA was expected not to be detectable, since the intronic portion of the LNA target sequence, the majority of the sequence of splice donor 3, is removed during nuclear splicing (Figure 2a).

To confirm this hypothesis, we designed an exon-exon-junction 5′ 6-FAM™ labeled SRSF6 LNA mixmer targeting the same 3′ end of exon 3, but instead of the downstream intronic sequence, it contains the 5′ end of the downstream exon 4 sequence which remains in the cytoplasmic mRNA after intron removal (SRSF6 ExJ 3/4 LNA, Figure 2d). Since we delivered the HIV targeting LNAs to HIV-1 infected Jurkat cells (Figure 1), we now gymnotically-delivered both SRSF6 LNA mixmers into this T-cell line. In line with the results obtained for HeLa cells, we again observed a cytoplasmic localization with a speckled distribution for both LNA mixmers (Figure 2e, and data not shown) confirming that at least within 48 h, LNA mixmers fail to enter the nucleus when gymnotically-delivered into HeLa or Jurkat cells.

Furthermore, as expected due to their cytoplasmic distribution, only gymnotically-delivered SRSF6 ExJ 3/4 LNA mixmer effectively induced SRSF6 mRNA degradation (Figure 2f, lane 3), substantiating our hypothesis that cytoplasmic-localized LNA mixmers induce degradation of mRNAs containing their target sequence.

### 2.3. The FAM-Labeled SRSF6 ExJ 3/4 LNA Mixmer Co-Localizes with GW-182 in HeLa and Jurkat Cells

Next, we wanted to analyze whether the “nakedly” delivered LNA mixmers and cytoplasmic GW-bodies known to contain RNA degrading enzymes co-localize. The reasons for addressing this question were first that glycine-tryptophan protein of 182 kDa (GW-182, GW-body marker) co-localized with gymnotically-delivered LNA gapmers targeting the ApoB or Bcl-2 mRNA in Huh-7 and HT 1080 cells [30,34]. Secondly, the gymnotically-delivered LNA mixmers used here also accumulated within the cytoplasm, and induced RNA degradation in HeLa and Jurkat cells which led to the assumption that the LNA mixmers might also co-localize with GW-bodies.

**Figure 3:**
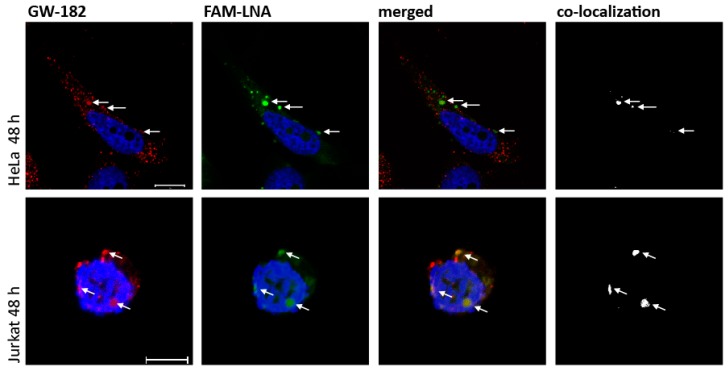
Co-localization of gymnotically-delivered 6-FAM™-SRSF6 ExJ 3/4 LNA mixmer with GW 182. HeLa or Jurkat cells were incubated with the 6-FAM™-SRSF6 ExJ 3/4 LNA. 48 h after gymnotic LNA delivery cells were fixed, permeabilized and incubated with a GW-182 antibody to detect GW-bodies. To monitor co-localization of the 6-FAM™-SRSF6 ExJ 3/4 LNA with GW-182, confocal laser scanning microscopy was performed. Red: GW-182; green: 6-FAM™-SRSF6 ExJ 3/4 LNA; white: Co-localization channel defining co-localized fluorescence. White bars: HeLa cells (10 µm), Jurkat cells (5 µm).

Therefore, we delivered the FAM-SRSF6 ExJ 3/4 LNA mixmer gymnotically, incubated HeLa or Jurkat cells for 48 h and after permeabilization and fixation, incubated the cells with a GW-182 antibody, and performed confocal laser scanning microscopy. Indeed, in both cell types, cytoplasmic co-localization of the LNA mixmer with GW-182 was observed to some extent (Figure 3, merged and co-localization, arrows). Although we detected several GW-bodies not co-localizing with the FAM-SRSF6 ExJ 3/4 LNA mixmer in HeLa cells (Figure 3, upper panel, red dots), co-localization was observed at cytoplasmic foci where the LNA mixmer accumulated within the cytoplasm (Figure 3, upper panel, FAM-LNA, merged and co-localization).

Co-localization could also be observed in Jurkat cells (Figure 3, lower panel, co-localization), though co-localization studies were more difficult due to their spherical shape and the unfavorable nucleus to cytoplasm ratio, which might explain why some foci appear to be in the nucleus.

In conclusion, these results suggest that enzymes localizing within GW-bodies might be involved in LNA mixmer-mediated degradation of mRNAs containing the LNA binding sequences.

### 2.4. Gymnotic Delivery of both LNA Mixmers, G_I3_-2 and ESE_tat_, Efficiently Interferes with Viral RNA Expression and HIV-1 Replication in Infected T-Cells

So far, our results demonstrated that gymnotically-delivered LNA mixmers induced degradation of mRNAs containing their target sequence within the cytoplasm, suggesting that the gymnotic delivery of the G_I3_-2 and ESE_tat_ LNA mixmers might interfere with HIV-1 replication.

Therefore, we next analyzed the impact of the gymnotically-delivered LNA mixmers on viral RNA expression and HIV-1 particle production. For this we infected different T-cell lines (Jurkat and PM1 cells) as well as primary T-cells (peripheral blood mononuclear cell (PBMC)) from a healthy donor, with the laboratory HIV-1 strain NL4-3 (MOI: 0.005). Six hours after infection we added the LNA mixmers in increasing concentrations (0.5, 1, 2 and 3 µM) to the cell culture medium. As an internal control we also applied the non SRSF6 RNA degrading SRSF6 D3 LNA (cf. Figure 2c,f) as this should not impair SRSF6 expression and thus not HIV-1 replication [15]. To analyze viral replication, we gymnotically-delivered LNA mixmers to HIV 1 infected cells and incubated the cells for 6 days. To measure the influence of the LNA mixmers on viral RNA expression we performed northern blot analysis using an HIV-1 exon 7 DIG-labeled probe. We performed immunoblot analysis detecting viral p24 Capsid (CA) protein (cellular and supernatant) to measure viral replication.

**Figure 4:**
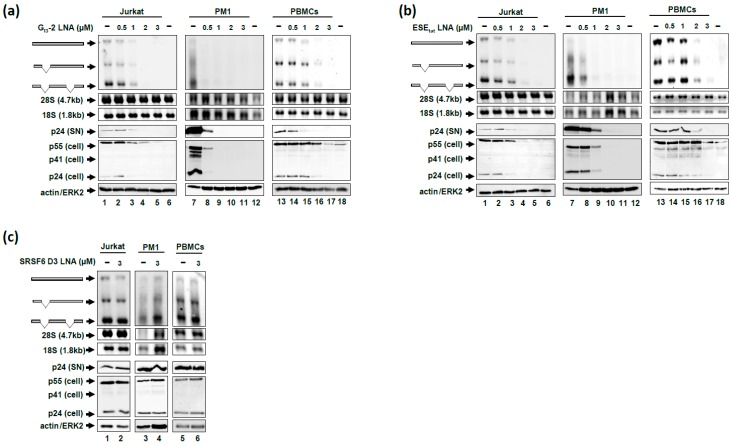
Interference of “nakedly” delivered G_I3_-2 and ESE_tat_ LNA mixmers with HIV-1 replication. Six hours after infection (NL4-3, MOI: 0.005) of Jurkat cells, PM1 cells or PBMCs with the laboratory strain NL4-3 the G_I3_-2 (**a**) and ESE_tat_ (**b**) LNA mixmers were added to the cell culture medium (0.5, 1, 2 and 3 µM). The “naked” delivery of the SRSF6 D3 LNA (**c**, 3 µM) served as the control. Total RNA and protein as well as virions from the supernatant were harvested six days post-delivery. The three viral mRNA classes were detected performing northern blot analysis using a DIG-labeled HIV-1 exon 7 probe. Cellular and supernatant p24 CA protein levels were determined by immunoblot analysis using an anti-p24 CA antibody. Detection of actin (Jurkat cells and PBMCs) or ERK2 (PM1 cells) served as loading control. In a and b lanes 6, 12 and 18 represent mock samples.

As shown in Figure 4, the “nakedly” delivered G_I3_-2 (Figure 4a) and ESE_tat_ (Figure 4b) LNA mixmers dramatically interfered with viral RNA expression and viral p24 CA expression in Jurkat and PM1 cells as well as in PBMCs. Both LNA mixmers, at a concentration of 1 or 2 μM, reduced the amount of all three viral RNA classes and p24 CA protein levels in Jurkat cells and in PBMCs (Figure 4a,b, cf. lane 1 with lanes 3–5 and lane 13 with lanes 15–17). The LNA-mediated antiviral effect was even stronger in PM1 cells affecting viral RNA expression and replication already at a concentration of 0.5 µM (Figure 4a,b, cf. lane 7 with lanes 8–11). On the contrary, when the SRSF6 D3 LNA was gymnotically-delivered at the highest concentration (3 µM) we could not observe effects neither on viral RNA expression nor HIV-1 replication (Figure 4c), indicating that not the LNA mixmers *per se* affect viral replication but the specific binding of the G_I3_-2 and ESE_tat_ LNA mixmers respectively to their target sequences.

In summary, both gymnotically-delivered LNA mixmers, G_I3_-2 and ESE_tat_, strongly interfered with HIV-1 RNA expression and replication in a low micromolar scale, which seems likely to be the result of the LNA-induced degradation of viral mRNA, demonstrating that unassisted delivered LNA mixmers efficiently interfere with HIV-1 replication in all host cells tested in this study.

### 2.5. The Antiretroviral Effect of the G_I3_-2 and ESE_tat_ LNA Mixmers as Well as a Cocktail Consisting of Both LNAs Lasts up to Nearly 14 Days in Jurkat Cells and in PBMCs

Because the unassisted delivery of G_I3_-2 and ESE_tat_ LNA mixmers displayed a strong antiretroviral effect, we next wanted to analyze for how long this inhibitory effect may last. For this, we set up a 14-day kinetic infecting Jurkat cells (NL4-3, MOI: 0.005) at day 1, and incubated the cells either with the G_I3_-2 or ESE_tat_ LNA mixmers at a concentration of 3 µM 6 h post infection. At day 6, 10 and 14 total RNA and protein were isolated and viral RNA expression and replication was monitored performing northern and immunoblot analysis.

**Figure 5:**
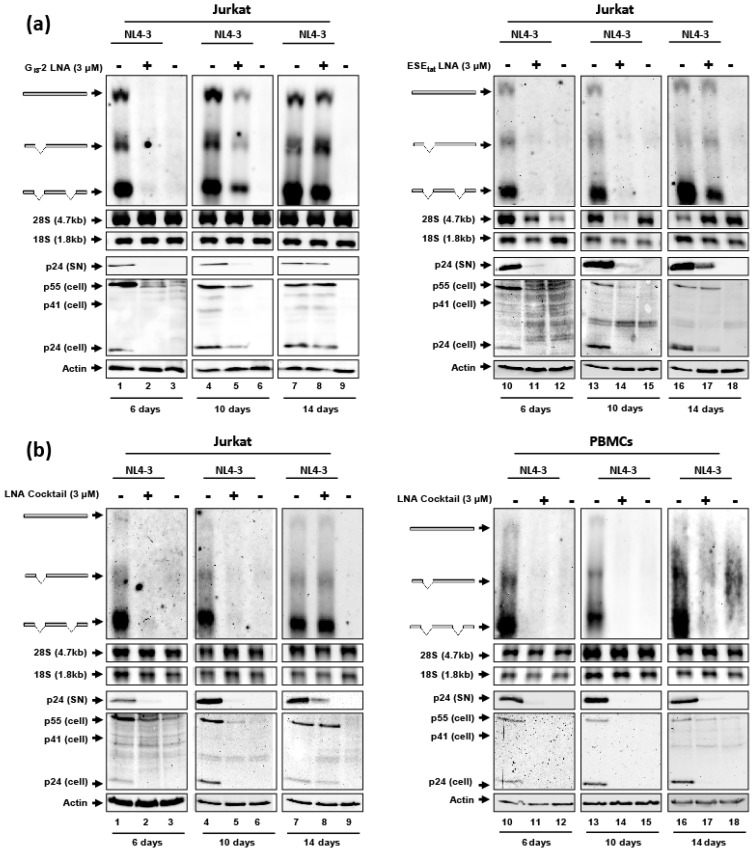
Antiretroviral effect of the gymnotically-delivered G_I3_-2 and ESE_tat_ LNA mixmers. Jurkat and PBMCs were infected with the laboratory HIV-1 strain NL4-3 (MOI: 0.005). Six hours post infection 3 µM of the G_I3_-2 or ESE_tat_ LNA (**a**) or both combined as an LNA cocktail (**b**, 1.5 µM of each LNA) were added to the cell culture medium. After 6 days, 10 days or 14 days total RNA and proteins, as well as virions from the supernatant were harvested. Viral mRNA classes were detected performing northern blot analysis using a DIG-labeled HIV-1 exon 7 probe. p24 CA protein levels were determined by immunoblot analysis using an anti-p24 CA antibody. Lanes 3, 6, 9, 12, 15 and 18 represent mock samples.

As shown in Figure 5a, after 6 and 10 days a clear impact on viral RNA and p24 CA protein expression for both LNAs could be observed (Figure 5a, 6 days and 10 days) and even after 14 days a slight influence was still visible (Figure 5a, 14 days), indicating that the LNA-mediated antiretroviral effect of the G_I3_-2 and ESE_tat_ LNA mixmer lasts at least 10 days.

In addition, because ART combines substances with different effects on the HIV-1 replication cycle to minimize viral escape mutants, we finally tested if combining both LNAs targeting different HIV-1 sequences to a LNA mixmer cocktail also displays a strong inhibitory effect on viral replication. For this analysis we infected Jurkat cells and PBMCs from a healthy donor (NL4-3, MOI: 0.005) and repeated the kinetic using 1.5 µM of each LNA mixmer.

As before, we observed a comparable interference with viral RNA and p24 CA protein expression after 6 and 10 days (Figure 5b, 6 days and 10 days). Furthermore, in PBMCs even 14 days after LNA cocktail application, viral replication was severely impaired (Figure 5b, PBMCs, 14 days) demonstrating the potential of LNA mixmers as antiretroviral compound.

## 3. Discussion

In this study we demonstrated that LNA mixmers delivered in the absence of any transfection reagent (gymnosis), targeting the HIV-1 splicing regulatory elements (SREs) G_I3_-2 and ESE_tat_ induced degradation of HIV-1 mRNA species containing their target sequence in HIV-1 infected T-cells. As a consequence, HIV-1 RNA expression and HIV-1 replication was affected, underlining the concept to use LNA mixmers as potential therapeutic compounds for the development of an antiretroviral therapy.

Since the G_I3_-2 and ESE_tat_ LNA mixmers as well as the SRSF6 D3 LNA mixmer localized within the nucleus and clearly influenced viral and cellular splicing after transfection (Figure 1b and Figure 2b) [13,15], it was surprising to find that not only LNA gapmers (LNAs at the 5′- and 3′ end and a DNA strand in the center of the antisense oligonucleotide (ASO)), but also LNA mixmers (mixed combination of LNA and DNA residues within the ASO), normally known to induce steric blocks, localize in the cytoplasm, and are able to induce RNA degradation after gymnotic delivery (Figure 1b and Figure 2f). This result suggested that the LNA mixmers intracellular localization (cytoplasmic vs. nuclear), and its effect on RNA expression (splice switching vs. RNA degradation), seems to depend on the mode of delivery. Although nuclear localization of gymnotically-delivered LNA gap/mixmers has been described [31,32,40], a predominantly cytoplasmic distribution after “naked” delivery of LNA gap/mixmers or other ASOs with phosphorothioate (PS) backbone seems to be the more prominent observation [30,34,41,42]. It was shown that chemical modifications of ASOs (e.g., PS-backbone, 2′-ribose-modification), as well as their sequences, can lead to interactions with intracellular proteins influencing the ASOs’ intracellular distribution and pharmacological actions [42,43,44]. Furthermore, the stress induced response complex (SIRC) mediates the translocation of ASOs, siRNAs or miRNAs to the nucleus, and could be induced either by transfection or other chemicals e.g., arsenite (As III) [45]. However, it was assumed that gymnotic delivery, contrary to lipofection, may not be a significant stressor to trigger SIRC-mediated nuclear transfer, resulting in a cytoplasmic localization of ASOs [45], explaining why the LNA mixmers used in this study accumulate in the cytoplasm and induce RNA degradation instead of affecting splicing within the nucleus. Indeed, we were not able to detect any ESE_tat_ LNA-mixmer-induced RNA degradation of viral target mRNAs after gymnotic delivery, when the cells were treated with transfection reagents at any time during the course of the experiment, excluding synergistic effects of combining both delivery methods (data not shown).

At the moment we are not aware of any off-target effects caused by the LNA mixmers used in this study. However, due to the fact that chemical modifications or sequences of ASOs and hence LNAs can e.g., lead to interactions with intracellular proteins, and thereby induce unspecific effects [42,43,44], further investigations are required to exclude such off-target effects for the G_I3_-2, ESE_tat_ and SRSF6 LNA mixmers.

So far, degradation of target mRNAs after gymnotic LNA delivery is only described for LNA gapmers whereas LNA mixmers are found to induce steric blocks and prevent protein binding, e.g., to induce switches within the splicing pattern [28,29]. Since LNA gapmers are known to induce RNase H1-mediated RNA degradation, which requires a gap of 7 to 10 neighboring deoxynucleotides within the LNA gapmer for noteworthy RNase H1 activity [46,47], it is highly unlikely that RNase H1 plays any role in an LNA-mixmer-induced RNA degradation. However, data obtained by Castanotto and colleagues [34] suggest that besides RNase H1, other proteins also involved in the cellular mRNA silencing machinery can be responsible for LNA gapmer-mediated RNA degradation.

The authors observed that gymnotically-delivered LNA gapmers displayed an identical cytoplasmic distribution as siRNAs delivered via transfection reagents, and that they co-localized with glycine-tryptophan protein of 182 kDa (GW-182), a finding we also observed when using LNA mixmers (Figure 3). Furthermore, they found that argonaute-2 (Ago-2), a protein involved in RNA interference (RNAi) which also interacts with GW-182, can bind LNA gapmers via its piwi/argonaute/zwille (PAZ) domain. In addition, they showed that after silencing the intracellular Ago-2 expression, the LNA gapmers’ function was impaired, indicating an involvement of Ago-2 in the LNA gapmers’ mode of action. Although the endonucleolytic cleavage activity of Ago-2 seems not to be involved in the LNA-gapmer-mediated RNA degradation, Ago-2 apparently functions as an escort protein, and is also a component of stress-induced response complex (SIRC) [34,45,48,49]. Therefore, and in agreement with the postulated hypothesis by Castanotto et al. [34], our gymnotically-delivered LNAs, targeting SREs or exon junctions, either as LNA gapmers or LNA mixmers, might be taken up by the cell via adsorptive and fluid-phase endocytosis. After membrane trafficking and transiting the late endosomal membrane, the LNAs bind to target mRNAs and induce translational steric blocks, and this results in the formation of GW-bodies. This, in a similar way described for cellular miRNAs, might be followed by the LNA-mediated RNA degradation, including recruitment of exo/endo-ribonucleases (e.g., XRN1), decapping enzymes (e.g., DCP1 and 2), and deadenylase-complexes (e.g., CCR4–NOT complex) [50,51,52]. However, it needs further and extended investigations to unravel the mechanism responsible for cytoplasmic LNA gapmer/mixmer-mediated RNA degradation.

Any new approach either affecting viral splicing, or inducing degradation of viral mRNA in order to suppress HIV-1 replication would be desirable. Both strategies result in an imbalance of HIV-1 RNA expression, and consequently interfere with HIV-1 replication. With regard to further development of such an anti-HIV-1 strategy, identification of additional viral RNA sequences as targets for LNAs would be beneficial. Since the LNA mixmers tested here accumulate within the cytoplasm and induce RNA degradation after gymnotic delivery, new LNA target sequences also available and accessible within the cytoplasm, are suitable. Furthermore, these viral sequences should be highly conserved among HIV-1 groups and subtypes. Several studies demonstrated that HIV-1 RNA sequences involved in viral gene expression and viral particle production are promising targets for ASOs. These include the viral primer binding site, the viral dimerization site, the viral major splice donor 1, the gag start codon, as well as the guanosine-adenosine-rich (GAR) splicing regulatory elements (SRE) and the trans-activation response element (TAR) [19,20,21,22]. Nevertheless, all these sequences were targeted via ASOs in cell-free in vitro or transfection experiments and therefore, it is of great interest to analyze if targeting these sequences with LNA mixmers also induce viral mRNA degradation, and are also suitable targets upon gymnotic delivery.

To further follow an LNA-based antiretroviral strategy, a combination of LNAs, which are most effective in inhibiting viral replication, would be an additional next step. In both, Jurkat cells and PBMCs, the G_I3_-2 and ESE_tat_ LNA-cocktail was very effective in inhibiting the viral replication (Figure 5b), indicating the potential of combining LNAs. The combination of different compounds interfering with different steps of the HIV-1 replication is successful with regard to viral escape in antiretroviral therapy (ART). Likewise, most of the aforementioned HIV-1 RNA sequences, as well as HIV-1 SREs and splice sites are highly conserved among different HIV subtypes. This brings along the advantage that an LNA cocktail, consisting of three to five efficient LNAs targeting different viral RNA sequences, may hamper the emergence of escape mutants. Although this has to be confirmed experimentally, an efficient LNA cocktail might be an alternative strategy for multi-drug resistant viruses and would be a promising addition or alternative to currently administered ART regimes.

## 4. Materials and Methods

### 4.1. Oligonucleotides

All locked nucleic acid (LNAs) antisense oligonucleotides used in this study were LNA/DNA mixmers of 16 nucleotide length with a phosphorothioate (PS) backbone. LNA mixmers were obtained from Qiagen/Exiqon (Hilden, Germany/Vedbaek, Denmark; see Table 1; red: Mismatch). Furthermore, the LNA content/positions within the LNA/DNA mixmers were also defined by Qiagen/Exiqon. Used Primer pairs were ordered from Metabion GmbH (see Table 2).

### 4.2. Cell Culture, Preparation of Virus Stocks, Infection Experiments and Gymnotic LNA Delivery

Jurkat and PM1 cells as well as PBMCs were cultured in RPMI1640 GlutaMax medium (Invitrogen, Carlsbad, Carlifornia, USA) containing 10% (*v*/*v*) fetal calf serum and 50 µg/mL each of both penicillin and streptomycin. PBMCs were additionally activated using 25 U/mL IL-2 (Roche, Basel, Switzerland). HeLa cells were maintained in Dulbecco’s high glucose modified Eagle’s medium (Invitrogen), supplemented with 10% (*v*/*v*) fetal calf serum, 50 μg/mL of penicillin and streptomycin each (Invitrogen).

Virus stocks were prepared as described in [13]. Multiplicity of infection (MOI) was determined by calculating the Tissue Culture Infection Dose of 50% (TCID50). 5 × 10^5^ Jurkat and PM1 cells or 1 × 10^6^ PBMCs were infected with the HIV-1 laboratory strain NL4-3 (MOI: 0.005) and, after six hours, cells were centrifuged, washed with PBS (Invitrogen) and resuspended in RPMI1640 GlutaMax medium (Invitrogen), containing only 10% (*v*/*v*) fetal calf serum. LNAs were then added to the cell culture medium at the indicated end concentration, respectively (gymnotic delivery). Jurkat cells, PM1 cells, and PBMCs were either incubated for 24 h, 48 h or 6, 10 or 14 days. The cell culture medium of PBMCs was supplemented with 25 U/mL IL-2 (Roche) every 3 days.

For gymnotic LNA delivery using HeLa cells, 1 × 10^4^ cells per well (six-well plate) were cultured and maintained in Dulbecco’s high glucose modified Eagle’s medium (Invitrogen) supplemented only with 10% (*v*/*v*) fetal calf serum. LNAs were then added to the cell culture medium at the indicated end concentration and the cells were incubated for 24 h, 48 h or 6 days.

### 4.3. Transfection of LNA Antisense Oligonucleotides

2.5 × 10^5^ HeLa cells per well were plated in a six-well plate and cultured in Opti-MEM medium containing 5% fetal calf serum. For LNA transfection, 80 nM (G_I3_-2 or ESE_tat_ LNA) or 100 nM (SRSF6 D3 LNA) of the LNAs were mixed with 4 µl Lipofectamine 2000 (Invitrogen) and Opti-MEN medium as described in [13,15,16].

### 4.4. RNA and Protein Isolation

Cells were centrifuged and washed with PBS (Invitrogen). Total RNA was isolated using acid guanidinium thiocyanate-phenol-chloroform as described in [53]. For protein isolation, cells were lysed in RIPA buffer (25 mM Tris·HCl pH 7.6, 150 mM NaCl, 1% NP-40, 1% sodium deoxycholate, 0.1% SDS, protease inhibitor cocktail (Roche)).

Furthermore, supernatants of infected cells were collected by sucrose centrifugation at 50,000× *g* for 1 h to quantify viral release by immunoblot analysis.

### 4.5. Immunoblot Analysis

Separation of proteins was performed by 15% SDS polyacrylamide gel electrophoresis. Subsequently, proteins were transferred to a nitrocellulose membrane (pore size, 0.45 µm; Protran, GE Healthcare, Chicago, Illinois, USA), and subjected to immunoblotting procedure. Membranes were probed with the respective primary antibodies: Sheep anti-p24 CA antibody (Aalto Bioreagents Ltd., Dublin, Ireland), mouse anti-β actin (Sigma-Aldrich, St. Louis, Missouri, USA) antibody, or anti-ERK2 antibody (Santa Cruz Biotechnology, Dallas, Texas, USA). After incubation with secondary antibodies (HRP-conjugated anti-mouse superclonal antibody (Invitrogen); HRP-conjugated anti-sheep antibody (Aalto Bioreagents Ltd.) the membrane was developed with ECL enhanced chemiluminescent reagent (GE Healthcare, Chicago, Illinois, USA).

### 4.6. RT-PCR-Analysis

For reverse transcription 2 µg of total RNA was subjected to cDNA synthesis. cDNA synthesis was performed for 1 h at 50 °C and 15 min at 72 °C by using 200 U Superscript III RNAse H Reverse Transcriptase (Invitrogen), 7.5 pmol oligo(dT)_12-18_ (Roche) as primer, 20 U of RNAsin (Promega, Fitchburg, Wisconsin, USA) and 10 mM of each deoxynucleoside triphosphate (Qiagen, Hilden, Germany). For semi-quantitative analysis cDNA was used as a template for PCR reactions. To detect the different mRNAs the following primer pairs were used: HIV-1 viral mRNAs (#1544/#3632); SRSF6 mRNA (#4933/#4934); ENO1 mRNA (#4907/#4908) or hGH mRNA (#1224/#1225). PCR products were separated on 10% nondenaturing polyacrylamide gels, stained with ethidium bromide, and visualized with an Imager (INTAS science imaging, Göttingen, Germany).

### 4.7. Northern Blot Analysis

Total-RNA was harvested from HIV-1 infected cells as described above and separated by gel electrophoresis. Subsequently, the RNA was capillary blotted overnight onto a nylon membrane, UV cross-linked and pre-hybridized with DIG Easy Hyb hybridization solution (Roche) for 2 h at 55 °C. The specific DIG-labeled probe (HIV-1 exon 7; #3387/#3388) was hybridized at 55 °C overnight. Finally, the membrane was washed, blocked and probed with anti-Digoxigenin-AP Fab fragments (Roche) followed by detection of the RNA bands using CDP-Star for chemiluminescent reactions (Roche).

### 4.8. Confocal Laser Scanning Microscopy

#### 4.8.1. Intracellular Localization of 6-FAM™-Labeled LNAs

To investigate the intracellular distribution of 6-FAM™-labeled LNAs after transfection, 1 × 10^4^ HeLa were seeded on cover slips in 24 well plates and were transfected with the FAM-SRSF6 D3 LNA as described above.

For monitoring the 6-FAM™-LNA localization within cells after gymnotic LNA delivery, 3 µM FAM-SRSF6 D3 or FAM-SRSF6 ExJ 3/4 LNA were added to the cell culture medium of 1 × 10^4^ HeLa or 5 × 10^5^ Jurkat cells. After 24 h (transfection) or 48 h (gymnotic delivery) of incubation, cells were washed three times with PBS, fixed with 4% paraformaldehyde (*w*/*v*) in PBS (10 min, RT), washed again two times with PBS and incubated with DAPI (1:5000 in PBS) to stain the nuclei. Cells were then washed two additional times with PBS. Cover slips with HeLa cells were fixed on glass slides with FluoromountG (Southern Biotech, Birmingham, Alabama, USA). The intracellular LNA localization was analyzed using a LSM780 confocal microscope (Zeiss, Oberkochen, Germany). Image analyses and processing was performed with the ZEN software (Zeiss).

#### 4.8.2. Co-Localization of Gymnotically-Delivered 6-FAM™-Labeled LNAs with GW-182

1 × 10^4^ HeLa cells seeded on cover slips or 5 × 10^5^ Jurkat cells were incubated with 3 µM FAM-SRSF6 ExJ 3/4 LNA for 48 h. Cells were fixed with 4% paraformaldehyde (*w*/*v*) in PBS (10 min, RT), permeabilized with 0.02% saponin (*v*/*v*) in PBS (15 min, RT) and blocked with 1% BSA (*v*/*v*), 0.002% saponin (*v*/*v*) in PBS for 60 min.

For staining GW-182, cells were incubated with a monoclonal mouse anti-GW-182 antibody (Abcam, Cambridge, UK) diluted 1:200 in 1% BSA (*v*/*v*), 0.0002% saponin (*v*/*v*) in PBS. After 2 h the antibody-solution was removed, cells were washed three times with 0.0002% saponin (*v*/*v*) in PBS for 5 min and incubated with Cy3 conjugated AffiniPure anti-mouse IgG diluted 1:300 1% BSA (*v*/*v*), 0.0002% saponin (*v*/*v*) in PBS for 45 min. This was followed by two times washing with 0.0002% saponin (*v*/*v*) in PBS for 5 min, staining of nuclei with DAPI (Invitrogen) (1:5000 in PBS) for 3 min and two additional washing steps with PBS. Co-localization of the FAM-SRSF6 ExJ 3/4 LNA with GW-182 was analyzed (including the co-localization channel to define co-localized fluorescence) using the ZEN software (Zeiss).

## 5. Conclusions

As demonstrated by the two FDA-approved antisense oligonucleotide-therapeutics for Duchenne muscular dystrophy (DMD) and spinal muscular atrophy (SMA), Eteplirsen and Nusinersen [24,25,27], we now have the tools to approach “undruggable” targets by specifically affecting RNA expression, and thereby expression of proteins. This includes viruses and thereby viral infections where RNA expression can be specifically affected utilizing the ASO technology in order to inhibit viral replication.

## Figures and Tables

**Table 1:** LNA/DNA mixmer oligonucleotides used in this work.

LNA Oligonucleotide	Sequence	Design-ID (Exiqon)	Cat. No Qiagen
G_I3_-2 [13]	TATGGCTCCCTCTGTG	164610	YCO0073444
ESE_tat_ [15]	TTCTTGCTCTCCTCTG	256589	YCO0073445
SRSF6 D3 (5’-end modiefied with 6-FAM™)	TACAAAACATACCTTT	319384	-
SRSF6 ExJ 3/4 (5’-end modiefied with 6-FAM™)	TCGCATAAAATCCTTT	548164	-
G_I3_-1-MM-control	TTTGGCTCACTCCGTG	240758	-

**Table 2:** DNA oligonucleotides used for semi-quantitative RT-PCR.

mRNA Type	Primer No.	Primer Sequence
HIV-1 exon1-4 mRNAs	#1544 (exon1)	5’ CTTGAAAGCGAAAGTAAAGC 3’
	#3632 (exon4)	5’ TGGATGCTTCCAGGGCTC 3’
HIV-1 exon7	#3387	5’ TTGCTCAATG CCACAGCCAT 3’
	#3388	5’ TTTGACCACT TGCCACCCAT 3’
SRSF6 mRNA	#4933	5’ GAGTTCGAGGACTCCCG 3’
	#4934	5’ TCTACTGCGGCTGCTCCT 3’
ENO1 mRNA	#4907	5’ CTGTGCCCAGTGGTGCT 3’
	#4908	5’ GACCTGAAGAACTCGGAGG 3’
hGH mRNA	#1224	5’ TCTTCCAGCCTCCCATCAGCGTTTGG 3’
	#1225	5’ CAACAGAAATCCAACCTAGAGCTGCT 3’

## Abbreviations

2′-MOE	2′-*O*-methoxyethyl
2′-OMe	2′-*O*-methyl
6-FAM™	6-Carboxyfluorescein
Ago-2	protein argonaute-2
ApoB	Apolipoprotein B
ART	antiretroviral therapy
ASO	antisense oligonucleotide
Bcl-2	Apoptosis regulator Bcl-2
CA	capsid
CCR4–NOT	carbon catabolite repressor 4- negative on TATA
D3	splice donor 3
DCP1/2	mRNA-decapping enzyme 1/2
DMD	Duchenne muscular dystrophy
ERK2	mitogen-activated protein kinase 1
ESEtat	exonic splicing enhancer tat
ExJ 3/4	exon junction exon3/exon4
FDA	U.S. Food and Drug Administration
Gapmer	LNAs at the 5′- and 3′ end and a DNA strand in the center of the ASO
GAR	guanosine-adenosine-rich exonic splicing enhancer
GI3-2	second G-run within HIV-1 intron 3
GW-182	glycine-tryptophan protein of 182 kDa
GW-body	cytoplasmic foci containing enzymes involved in RNA degradation and translational repression
HCV	hepatitis C virus
HIV-1	human immunodeficiency virus type 1
hnRNP	Heterogeneous nuclear ribonucleoproteins
IL-2	Interleukin-2
LNA	Locked nucleic acid
miRNA	microRNA
Mixmer	mixed combination of LNA and DNA residues within the ASO
PAZ domain	Piwi/Argonaute/Zwille domain
PBMC	peripheral blood mononuclear cell
PMO	phosphorodiamidate morpholinos
PS	phosphorothioate
RNAi	RNA interference
RRE	Rev responsive element
SA3	splice acceptor 3
SIRC	stress-induced response complex
siRNA	small interfering RNA
SMA	spinal muscular atrophy
SR	serine and arginine-rich protein
SRE	splicing regulatory element
SRSF6	serine/arginine-rich splicing factor 6
SSO	splice-switching oligonucleotide
TAR	trans-activation response element
U1 snRNA	U1 small nuclear ribonucleic acid
WHO	World Health Organization
XRN1	5’-3’ exoribonuclease 1
